# Topical 1.5% ruxolitinib cream for facial refractory segmental vitiligo: A retrospective single-center study

**DOI:** 10.1016/j.jdin.2025.08.016

**Published:** 2025-10-03

**Authors:** Hequn Huang, Dawei Duan, He Huang, Xianfa Tang, Caihong Zhu, Yaohua Zhang, Min Li, Yujun Sheng, Leihong Xiang, Yong Cui, Youwen Zhou, Xuejun Zhang, Bo Zhang

**Affiliations:** a Department of Dermatology, The First Affiliated Hospital, Anhui Medical University, Hefei, Anhui, China; b Department of Dermatology, The Fourth Affiliated Hospital of Soochow University (Suzhou Dushu Lake Hospital Medical Center of Soochow University), Suzhou, China; c Department of Dermatology, Institute of Dermatology, Huashan Hospital, Fudan University, Shanghai, China; d Department of Dermatology, China-Japan Friendship Hospital, Beijing, China; e Department of Dermatology and Skin Science, University of British Columbia, Vancouver, Canada; f Dermatology Center in Boao Super Hospital, Oionghai, China

**Keywords:** efficacy, Janus kinase inhibitor, refractory, ruxolitinib cream, segmental vitiligo

*To the Editor:* Segmental vitiligo (SV) is a subtype of vitiligo characterized by unilateral and localized skin depigmentation, accounting for 5% to 27.9% of patients with vitiligo. SV generally manifests at an earlier age and exhibits poorer response to treatment compared with non-SV (NSV). Currently, no standardized therapeutic approach exists. Recently, Janus kinase inhibitors, especially topical 1.5% ruxolitinib cream (which selectively inhibits Janus kinase 1 or Janus kinase 2), have emerged as promising treatment options for vitiligo. Topical 1.5% ruxolitinib cream has received regulatory approval in the United States for NSV, based on the results of phase 3 clinical trials.^1^ However, research on the treatment of SV with ruxolitinib cream is limited to case reports.^2^ In the phase 2 clinical study of ruxolitinib cream in the treatment of vitiligo, although 11 cases of SV were involved, data from segmental trials were not separately presented.^3^

Here, we report a single-center case series involving 40 patients (25 in the progressive stage and 15 in the stable stage) with refractory facial SV (mean age, 26.90 ± 10.12 years) who had failed at least 12 weeks of multiple conventional treatments and were subsequently treated with 1.5% ruxolitinib cream. Facial SV was diagnosed clinically by 2 independent observers (H.H. and D.D.) based on localized depigmented macules arranged in a particular configuration. After excluding contraindications and obtaining the patient’s complete informed consent, they received topical 1.5% ruxolitinib cream twice daily for at least 24 weeks. The detailed clinical information of these patients is presented in Table I.

**Table I:** Demographic and clinical characteristics of patients with vitiligo

Characteristics	(*N* = 40)
Gender, *n* (%)	
F	23 (57.5)
M	17 (42.5)
Age, y	26.90 ± 10.12
Age of first onset, y	23.28 ± 10.99
Disease duration, y	4.91 ± 7.54
Family history, *n* (%)	
Yes	6 (15)
No	34 (85)
Comorbid conditions, *n* (%)	
SLE	1 (2.5)
DI	1 (2.5)
TD	1 (2.5)
TD and DI	1 (2.5)
Previous therapies, *n* (%)	
TCS	38 (95)
TCI	36 (90)
NB-UV-B	30 (75)
OS	13 (32.5)
JAKi	2 (5)
ETS	2 (5)
VIDA score, *n* (%)	
0	14 (35)
1	5 (12.5)
2	5 (12)
3	7 (17.5)
4	9 (22.5)
Term, *n* (%)	
Progressive	25 (62.5)
Stable	15 (37.5)

VIDA score: 0 points for stable stage, 1 to 4 points for progressive stage.

*DI*, Diabetes; *ETS*, epidermal transplantation surgery; *F*, female; *JAKi*, Janus kinase inhibitors; *M*, male; *NB-UV-B*, narrow band-UV-B; *OS*, oral steroid; *SLE*, systemic lupus erythematosus; *TCI*, topical calcineurin inhibitors; *TCS*, topical corticosteroids; *TD*, thyroid disease; VIDA, vitiligo disease activity.

The mean baseline Vitiligo Area Scoring Index (VASI) score of all patients was 0.17 ± 0.15. At week 24, a total of 18 patients (*n* = 45%; 95% CI, 0.84-0.94) achieved the primary end point of ≥75% improvement in VASI (VASI75). The proportion of patients reaching VASI75 was significantly greater in the progressive-stage group (16 patients, 64%) compared to the stable-stage group (2 patients, 13%; *P* < .05) (in Fig 1). Furthermore, the mean VASI scores improved to 0.10 ± 0.11 at week 12 and further to 0.05 ± 0.06 at week 24, corresponding to 42.4% and 71.3% reductions from baseline, respectively. This difference in improvement rates between weeks 12 and 24 was statistically significant (*P* < .01) (detailed information regarding treatment responses is shown in Fig 1 and Supplementary Table I, available via Mendeley at https://data.mendeley.com/datasets/8pp7ps3mnk/1). Although our VASI 75 rates at 24 weeks exceeded those reported for traditional SV medical therapies (topical corticosteroids or topical calcineurin inhibitors),^4^ they remained below the results of ruxolitinib cream in Chinese patients with NSV.^5^ These comparisons require cautious interpretation because of differing study designs and populations, and the small sample size may introduce selection bias. No serious adverse reactions were observed in this study; only 3 patients had mild erythema, and 2 had mild acne (detailed information regarding treatment responses is shown in Supplementary Table II, available via Mendeley at https://data.mendeley.com/datasets/8pp7ps3mnk/1).

**Fig 1:**
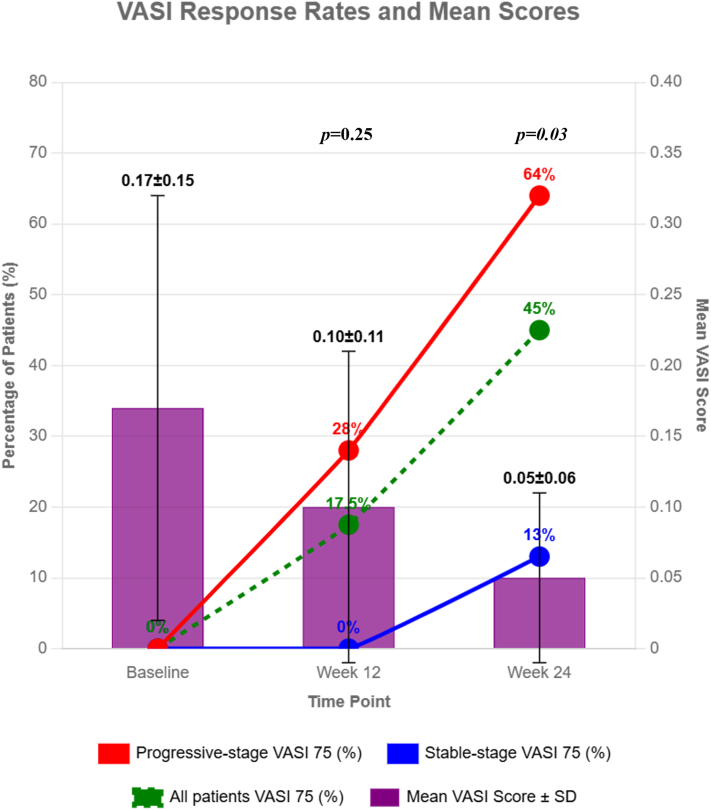
Statistical significance (*P*-values) of treatment outcomes at weeks 12 and 24. The achievement of VASI75 rates at 12 and 24 weeks in the progressive-stage group (*red line*), stable-stage group (*blue line*), and all-patient group (*green line*). Mean VASI scores (*purple bar*). At week 24, the VASI75 achievement rate showed a statistically significant difference between the progressive-stage group and the stable-stage group (*P* = .03).

In summary, our findings indicate that topical ruxolitinib cream demonstrated significant efficacy with a favorable safety profile. Patients showed a trend toward better repigmentation response during the progressive stage compared to the stable stage. However, the number of cases in this series is too small to draw definitive conclusions. More studies are needed in the future to fully evaluate the usefulness and safety of this treatment for SV.

## Conflicts of interest

None disclosed.

## References

[1] Rosmarin D., Passeron T., Pandya A.G., TRuE-V Study Group (2022). Two phase 3, randomized, controlled trials of ruxolitinib cream for Vitiligo. N Engl J Med.

[2] Meister H.M., Lebwohl M., Silverberg N. (2024). Case series of topical 1.5% ruxolitinib cream for pediatric vitiligo. JAAD Case Rep.

[3] Rosmarin D., Pandya A.G., Lebwohl M. (2020). Ruxolitinib cream for treatment of vitiligo: a randomised, controlled, phase 2 trial. Lancet.

[4] Kathuria S., Khaitan B.K., Ramam M., Sharma V.K. (2012). Segmental vitiligo: a randomized controlled trial to evaluate efficacy and safety of 0.1% tacrolimus ointment vs 0.05% fluticasone propionate cream. Indian J Dermatol Venereol Leprol.

[5] Huang H., Sheng Y., Li M. (2025). Effectiveness and safety of ruxolitinib cream in Chinese patients with nonsegmental vitiligo. J Am Acad Dermatol.

